# Identification of key genes for triacylglycerol biosynthesis and storage in herbaceous peony (*Paeonia lactifolra* Pall.) seeds based on full-length transcriptome

**DOI:** 10.1186/s12864-024-10513-w

**Published:** 2024-06-15

**Authors:** Huajie Xu, Miao Li, Di Ma, Jiajun Gao, Jun Tao, Jiasong Meng

**Affiliations:** 1 College of Horticulture and Landscape Architecture, Yangzhou University, Yangzhou, 225009 China; 2 Joint International Research Laboratory of Agriculture and Agri-Product Safety, the Ministry of Education of China, Yangzhou University, Yangzhou, 225009 China

**Keywords:** *Paeonia lactiflora* ‘Hangshao’, Full-length transcriptome, PacBio Iso-Seq, Triacylglycerol, Oleosin, Diacylglycerol acyltransferase

## Abstract

**Background:**

The herbaceous peony (*Paeonia lactiflora* Pall.) is extensively cultivated in China due to its root being used as a traditional Chinese medicine known as ‘Radix Paeoniae Alba’. In recent years, it has been discovered that its seeds incorporate abundant unsaturated fatty acids, thereby presenting a potential new oilseed plant. Surprisingly, little is known about the full-length transcriptome sequencing of *Paeonia lactiflora*, limiting research into its gene function and molecular mechanisms.

**Results:**

A total of 484,931 Reads of Inserts (ROI) sequences and 1,455,771 full-Length non-chimeric reads (FLNC) sequences were obtained for CDS prediction, TF analysis, SSR analysis and lncRNA identification. In addition, gene function annotation and gene structure analysis were performed. A total of 4905 transcripts were related to lipid metabolism biosynthesis pathway, belonging to 28 enzymes. We use these data to identify 10 oleosin (OLE) and 5 diacylglycerol acyltransferase (DGAT*)* gene members after de-redundancy. The analysis of physicochemical properties and secondary structure showed them similarity in gene family respectively. The phylogenetic analysis showed that the distribution of OLE and DGAT family members was roughly the same as that of Arabidopsis. Quantitative real-time polymerase chain reaction (qRT–PCR) analyses revealed expression changes in different seed development stages, and showed a trend of increasing and then decreasing.

**Conclusion:**

In summary, these results provide new insights into the molecular mechanism of triacylglycerol (TAG) biosynthesis and storage during the seedling stage in *Paeonia lactiflora*. It provides theoretical references for selecting and breeding oil varieties and understanding the functions of oil storage as well as lipid synthesis related genes in *Paeonia lactiflora*.

**Supplementary Information:**

The online version contains supplementary material available at 10.1186/s12864-024-10513-w.

## Introduction

In China, herbaceous peony (*Paeonia lactiflora* Pall.) is a famous flower with excellent ornamental value, it belongs to paeonia, paeoniaceae. There is only one genus of peonies in the family paeoniaceae, among which the herbaceous peony is widely loved for its large and beautiful flowers, it symbolizes wealth, prosperity and happiness. There are eight species of herbaceous peony in China (Supplementary table: Table S1), among which *Paeonia lactiflora* is the most widely spread throughout the country [1]. As a member of herbaceous peony, ‘Hangshao’ is mainly cultivated in areas such as Zhejiang, Sichuan and Anhui due to its medicinal value and clearly characterized by white or pink single petals. In recent years, with the recognition of tree peony as a new type of oil resources [2], the research on the oil function of herbaceous peony in the same family and genus has been increasingly emphasised. Additionally, the oil yield of ‘Hangshao’ seeds tended to increase with seed development [3], and it has been shown that the seed yield of ‘Hangshao’ at maturity is higher than the oil peony [4]. It is expected to be developed as a new oil plant due to the seed of ‘Hangshao’ has a high fruiting rate, oil content and unsaturated fatty acid content [5]. Consequently, ‘Hangshao’ was used the material of transcriptome sequencing to lay the foundation for exploring the molecular mechanism of lipid synthesis in ‘Hangshao’. Unfortunately, no high-quality genome sequence is available for reference in herbaceous peony, and thus transcriptome sequencing offers a valuable alternative for gene mining and functional characterization [6, 7].

The oil of oil-bearing crop is mainly distributed in seeds, and the formation and accumulation of oil in seeds, mainly include fatty acid synthesis, triacylglycerol (TAG) assembly and oil body formation, involving a series of physiological and biochemical processes [8–11]. Lipids are mainly stored as the form of triacylglycerols in seed oil bodies, which are generally liquid matrices of triacylglycerols on the inside and a single layer of phospholipids on the outside, and several binding proteins are embedded in this semi-unit membrane. Among them, oleosin (OLE) plays important roles in the formation and stability of oil body, that is the earliest and most abundant protein found in the oil binding protein, while diacylglycerol acyltransferase (DGAT) directly involved in TAG synthesis [12]. The function of *DGAT* in TAG synthesis has been validated in peanut and *oleaginous yeast* [13]. It was shown that heterologous expression of *AhDGAT1-1* and *AhDGAT1-2* in yeast restored the ability of mutant yeast to lipids synthesis, and that heterologous expression of *AhDGAT2a* and *AhDGAT2b* in *Escherichia coli* significantly increased the fatty acid content of *E. coli* [14]. Excessive expression of the *OLE* gene can prevent oil melting to maintain the size of oil body, as in Arabidopsis *AtOLE1* mutants, late seed stage leads to oil melting due to the lack of oil proteins. The product becomes larger, making the developing seeds more sensitive to low temperatures [15], indicating OLE can be used as a key protein for seed frost resistance [16]. In addition, the *BnOLE* gene promotes transgenic Arabidopsis seeds development and increased oil content [17], oil proteins can be used as binding sites for lipases, mobilizing for the storage of TAG to provides energy for seed germination [18]. The *OLE* and *DGAT* gene plays important roles in promoting seed development, regulates oil morphology and increases seed oil content quantity. However, studies on *OLE* and *DGAT* in herbaceous peony seeds have been reported rarely.

Currently, three generations of transcriptome sequencing enables sequencing reads in the size of thousands of bases [19], showing more RNA molecules [20], which have been applied to investigate full-length transcriptomes of different species, such as wheat [21], salvia [22], sorghum [23], maize [24], sugarcane [25], perennial rye grass [26],Chinese cabbage [27], etc. Combining RNA-Seq, Iso-Seq and proteomic identification methods, Zhu investigated the mechanism of Alternative Splicing (AS) in the model plant Arabidopsis after treatment with abscisic acid (ABA) [28]. Studies have compared transcriptional differences in different parts of bamboo using Iso-Seq, revealing the growth and development mechanisms of underground rhizomes in *Phyllostachys heterocycla*. In conclusion, the three generation transcriptome sequencing technology has been widely applied, especially advancing research in the field of plant. The purpose of this study is to apply PacBio full-length sequencing to provide a basis for in-depth understanding of the *OLE* and *DGAT* gene family in *P. lactiflora*, this paper mainly collected young leaves, roots, stems, seeds, flowers and stamens for full-length transcriptome sequencing, and analyzed the *P. lactiflora* ‘Hangshao’ transcriptome, will provide valuable genetic resources for further study of the evolutionary and biological functions of *Paeonia lactiflora.*

## Results

### Full-length transcriptome sequencing with SMRT analysis

Through the PacBio Sequel platform, we co-sequenced a sample and established a total of PacBio IsoSeq library which yielded 554,117 polymerase reads (41.35 GB), in total, 170,904 genes were detected. The ROI sequence was extracted from the original sequence according to the condition that full passes ≥ 0 and the sequence accuracy ≥ 0.75. Then calculate the offline date, the number of ROI in the library, the number of bases for the ROI, and the Mean Read Length of Insert sequence. Based on the test results, a total of 484,931 ROI sequences were generated in SMRT cell sequencing, and the Mean Read Quality of Insert was above 97% (Supplementary table: Table S2).

**Fig. 1:**
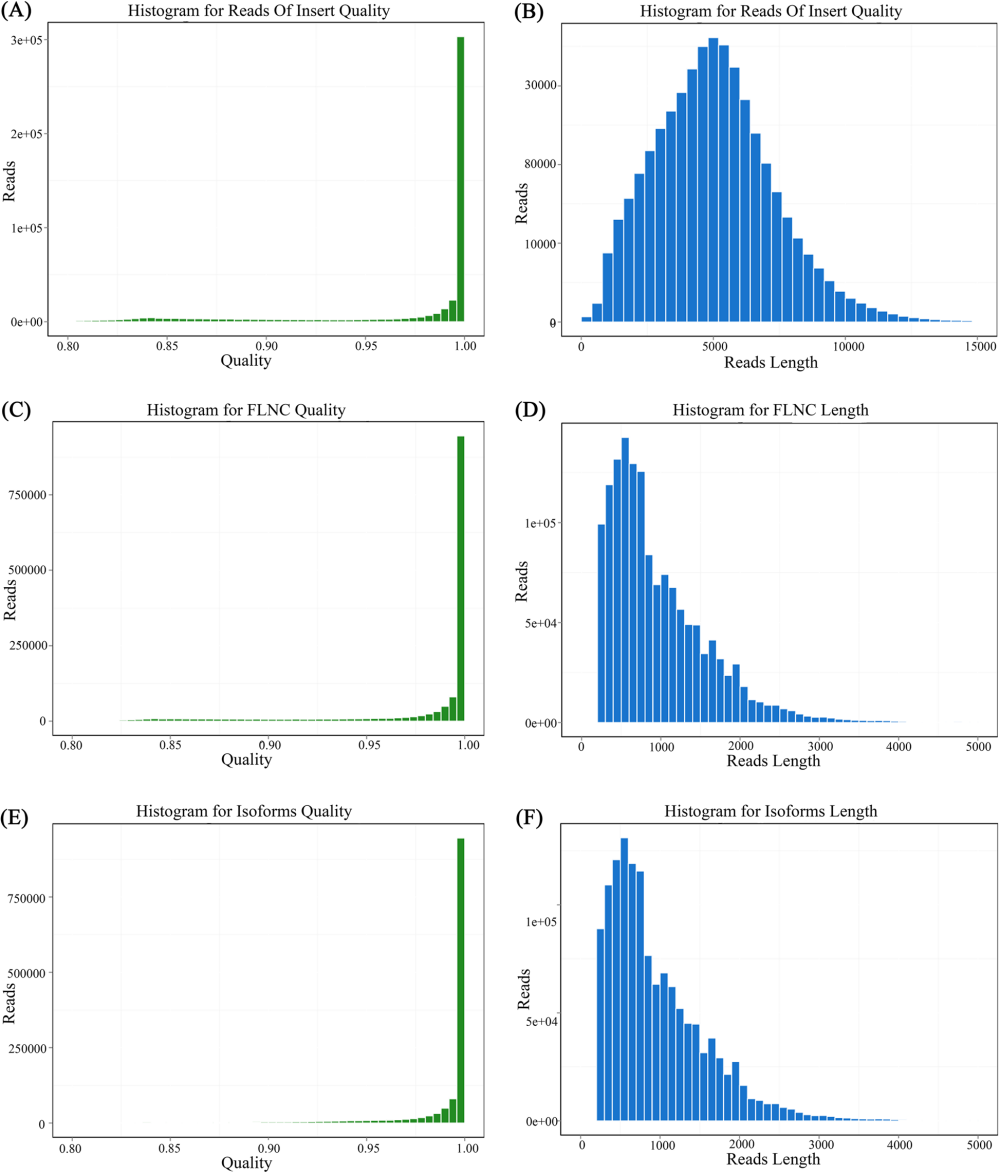
Quality and length distribution of Reads of insert (ROI), full-length non-chimeric (FLNC) and Isoforms. **A**, **B** Quality and length distribution of ROI. **C**, **D** Quality and length distribution of FL. **E**, **F** Quality and length distribution of Isoforms

By screening short fragments < 300 bp, sequences containing both 3’primers and 5’ primers with the presence of poly A tail before the 3’primers were defined as full-length sequences. After further screening and analysis, 1,455,771 full-length non-chimeric (FLNC) reads were obtained, and the peak movements of the two charts were consistent and in line with expectations (Fig. 1). Furthermore, CD-Hit-V4.6.7 was used to remove redundancy for subsequent analysis. 1,335,148 transcripts were obtained and the common gene samples are 282,635. The total length was 319,979,564 bp, the maximum length of the 282,635 genes was 42,047 bp, the minimum was 200 bp, and the GC content was 41.33% (Table 1). The obtained de-redundant transcripts were sorted by length, and the resulting N50 and N90 statistics were 1,514 and 584 bp, respectively. Quality control of raw reads was conducted with FASTP to filter low-quality data and clean the obtained reads. All data met the requirements and could be conducted in subsequent tests.

**Table 1:** Summary of the final transcript sequence after de-redundancy

Total number	Total length (bp)	Maximum Length (bp)	Minimum Length (bp)	N50 Length (bp)	N90 Length (bp)	GC Content (%)
282,635	319,979,564	42,047	200	1,514	584	41.33

### Functional annotation of genes

**Fig. 2:**
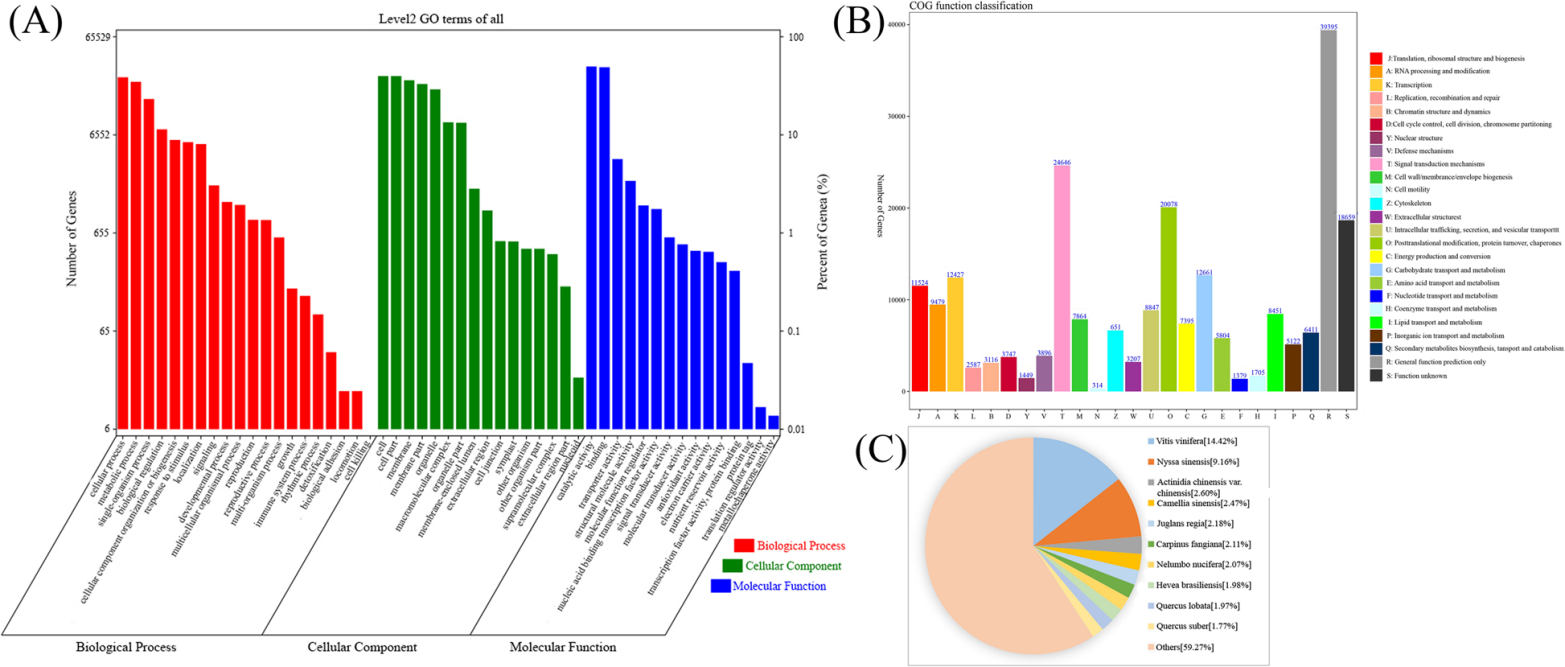
Function annotation of transcripts. **A** Distribution of GO terms for all annotated transcripts in biological process, cellular component and molecular function.** B** The COG function classification of consensus sequence. **C** The Nr Homologous species distribution

The GO annotation system consists of three main branches, along with biological processes, molecular functions, and cellular components. After GO annotation of the obtained isoforms, 51 biological function annotations were obtained under three categories. In the biological process, the cellular process, metabolic process and single-organism process were among the 20 terms that accounted for high proportions. In the cellular component, the cell, cell part, membrane, membrane part and organelle were among the 16 terms that accounted for high proportions. In the molecular function, the catalytic activities and binding were among the 15 terms that accounted for high proportions (Fig. 2A).

Furthermore, we annotated the full-length transcriptome with the COG database, and the 166,100 annotated genes were associated with 25 processes such as RNA processing and modification, among which the Signal transduction mechanisms (24,646), Posttranslational modification, protein turnover, chaperones (20,078), and the General function prediction only (39,395) were most abundant, the Lipid transport and metabolism was annotated 8,451, these transcripts associated with lipid metabolism may be involved in the biosynthesis of unsaturated fatty acids and lipid metabolism pathways of the herbaceous peony, while Cell motility (314) and Nucleotide transport and metabolism (1,379) were less abundant (Fig. 2B).

We have submitted the final polished consensus mRNA sequence to the NCBI. Blast software compares non-redundant transcripts with Nr, Nt SwissProt, GO, COG, Pfam, and KEGG databases. A total of 282,635 transcript annotation information points were obtained. Among these isoforms, 210,927 were observed in Nr (74.64%), 174,649 in Nt (61.79%), 161,615 in SwissProt (57.18%), 166,100 in COG (58.77%), 131,865 in Pfam (46.66%), 165,253 in GO (58.47%), and 164,473 in KEGG (58.19%) (Table 2). We looked for homologous species by sequence alignment. The permutation of transcripts among the Nr 210,972 isoforms shows the largest distribution of transcripts in *Vitis vinifera* (14.42%), followed by *Nyssa sinensis* (9.16%) and *Actinidia chinensis* (2.60%) (Fig. 2C).

**Table 2:** Transcript function annotation statistics

Annotated databases	Isoform number	Percentage
Nr	210,972	74.64%
Nt	174,649	61.79%
SwissProt	161,615	57.18%
KEGG	164,473	58.19%
COG	166,100	58.77%
Pfam	131,865	46.66%
GO	165,253	58.47%
All annotated	282,635	100%

### Gene structure analysis

**Fig. 3:**
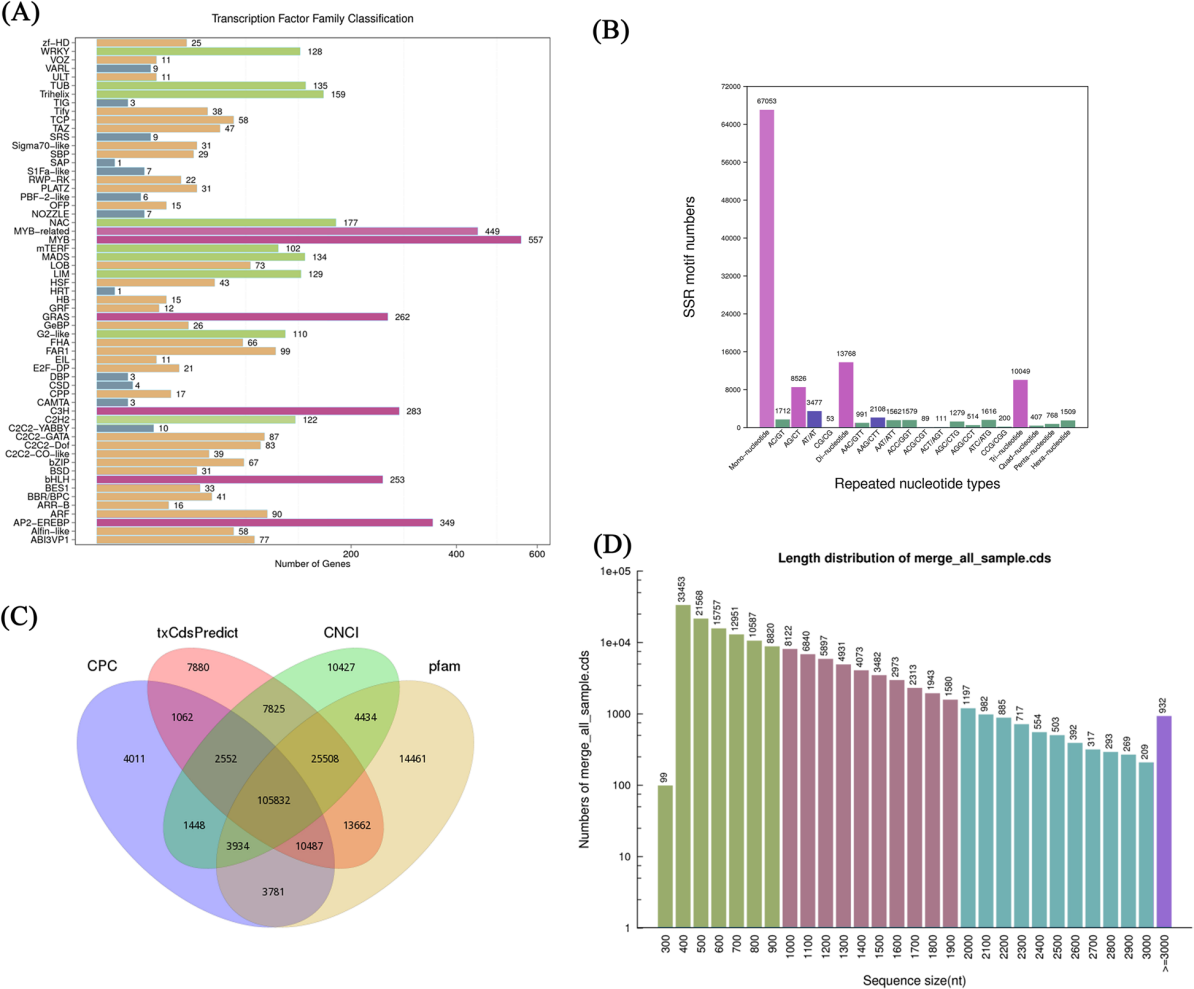
Gene structure analysis of transcripts. **A** Transcription factor (TF) analysis. **B** The simple sequence repeats (SSR) analysis. **C** Venn diagram of lncRNAs prediction. **D** The coding sequence (CDS) length distribution

Firstly, we conducted transcriptome-wide identification of transcription factor families from *Paeonia lactiflora* full-length transcriptome using animalTFDB2.0 [29]. In this study, a total of 4,735 transcripts encoding 59 types of TFs were identifed through blasting with PlnTFDB database. The most abundant transcription factor families are MYB (557), MYB-related (449), AP2-EREBP (349), C3H (283), GRAS (262) and bHLH (253) (Fig. 3A). Analysis of the transcription factor family of *Paeonia lactiflora* 'Hangshao' allowed a deeper understanding of their interactions with target genes and gene regulatory networks, laying a solid foundation for later studies.

Additionally, full-length transcriptome has been helpful for marking discovery of simple sequence repeats (SSR). MISA (http://pgrc.ipkgatersleben.de/misa/misa.html) was used to identify SSRs. The primary type of SSRs (> 6,4000 SSRs) was mono-nucleotide, followed with di-nucleotide (~ 10,000SSRs) (Fig. 3B). We found that mono-, di- and Tri- nucleotide repeats (77.42%) were the dominant motifs for SSR loci, with mono- and di- nucleotide repeat types accounting for 68.86% of the overall number of SSR motifs, which may indirectly account for the complexity and diversity in ‘Hangshao’.

Furthermore, we used four methods to predict long non-coding RNAs (lncRNAs) in the full-length transcriptome. The lncRNA were predicted by CNCI [30], txCdsPredict [31], CPC [31], and Pfam [32]. A total number of 217,304 lncRNAs were found in the full-length transcriptome. A total of 133,107 lncRNAs, 174,808 lncRNAs, 161,960 lncRNAs, 182,099 lncRNAs were found using CPC, txCdsPredict, CNCI, Pfam, respectively. Subsequently, we conducted an upset plot analysis of lncRNAs predicted by the four kinds of software and found that a total of 105,832 lncRNAs existed simultaneously (Fig. 3C).

The gene structure analysis was conducted based on CDS prediction, SSR analysis, lncRNA prediction, and transcriptional factor analysis. The coding sequence (CDS) is a sequence that encodes a protein product. Predicting the CDS of a protein is helpful for preliminary genetic analysis and is the basis for subsequent analysis of the protein structure. CDS prediction analysis was conducted using ANGEL software [33]. In CDS prediction, the CDS length of over 90% is < 3,000 bp. A total of 152,639 CDS were predicted, mainly between 400 and 3000 bp in length (Fig. 3D).

### Identification of enzyme genes in lipid metabolism biosynthesis

**Fig. 4:**
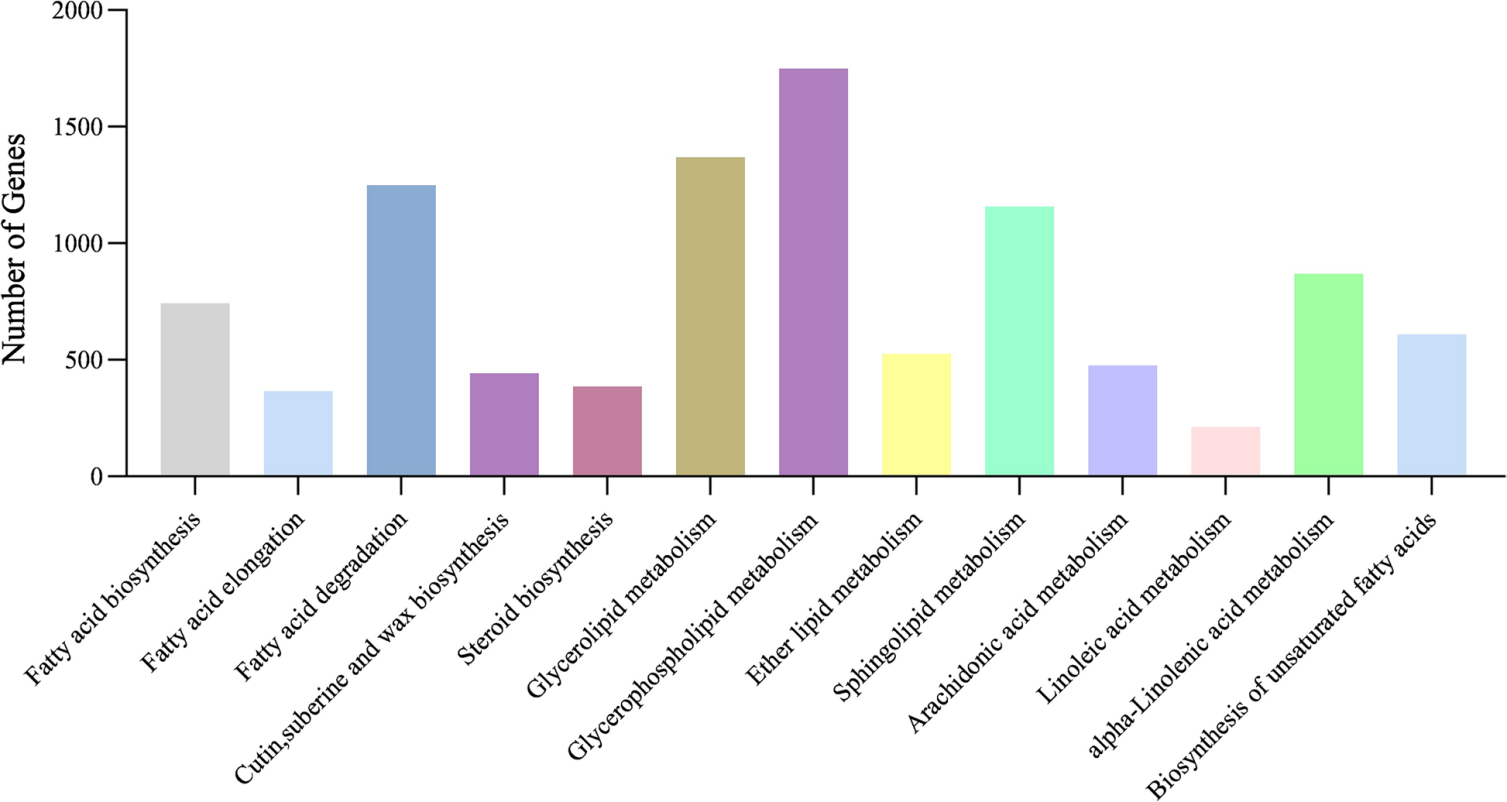
Lipid metabolism pathway related genes

Based on the functional annotations of the genes, we identified 10,151 transcripts associated with lipid metabolism (Fig. 4). These transcripts were associated with 13 metabolic pathways: fatty acid biosynthesis (742 transcripts), fatty acid elongation (366 transcripts), fatty acid degradation (1,249 transcripts), cutin, suberine and wax biosynthesis (443 transcripts), steroid biosynthesis (385 transcripts), glycerolipid metabolism (1,369 transcripts), glycerophospholipid metabolism (1,749 transcripts), ether lipid metabolism (525 transcripts), sphingolipid metabolism (1,157 transcripts), arachidonic acid metabolism (476 transcripts), linoleic acid metabolism (212 transcripts), alpha-linolenic acid metabolism (869 transcripts), biosynthesis of unsaturated fatty acids (609 transcripts). Of these 10,151 transcripts, 4,905 were associated with the biosynthesis of unsaturated fatty acids and oil accumulation, including fatty acid biosynthesis (474 transcripts), fatty acid elongation (362 transcripts), biosynthesis of unsaturated fatty acids (3,091 transcripts), triacylglycerol (TAG) biosynthesis (616 transcripts) and lipid storage (362 transcripts) (Supplementary table: Table S3).

**Fig. 5:**
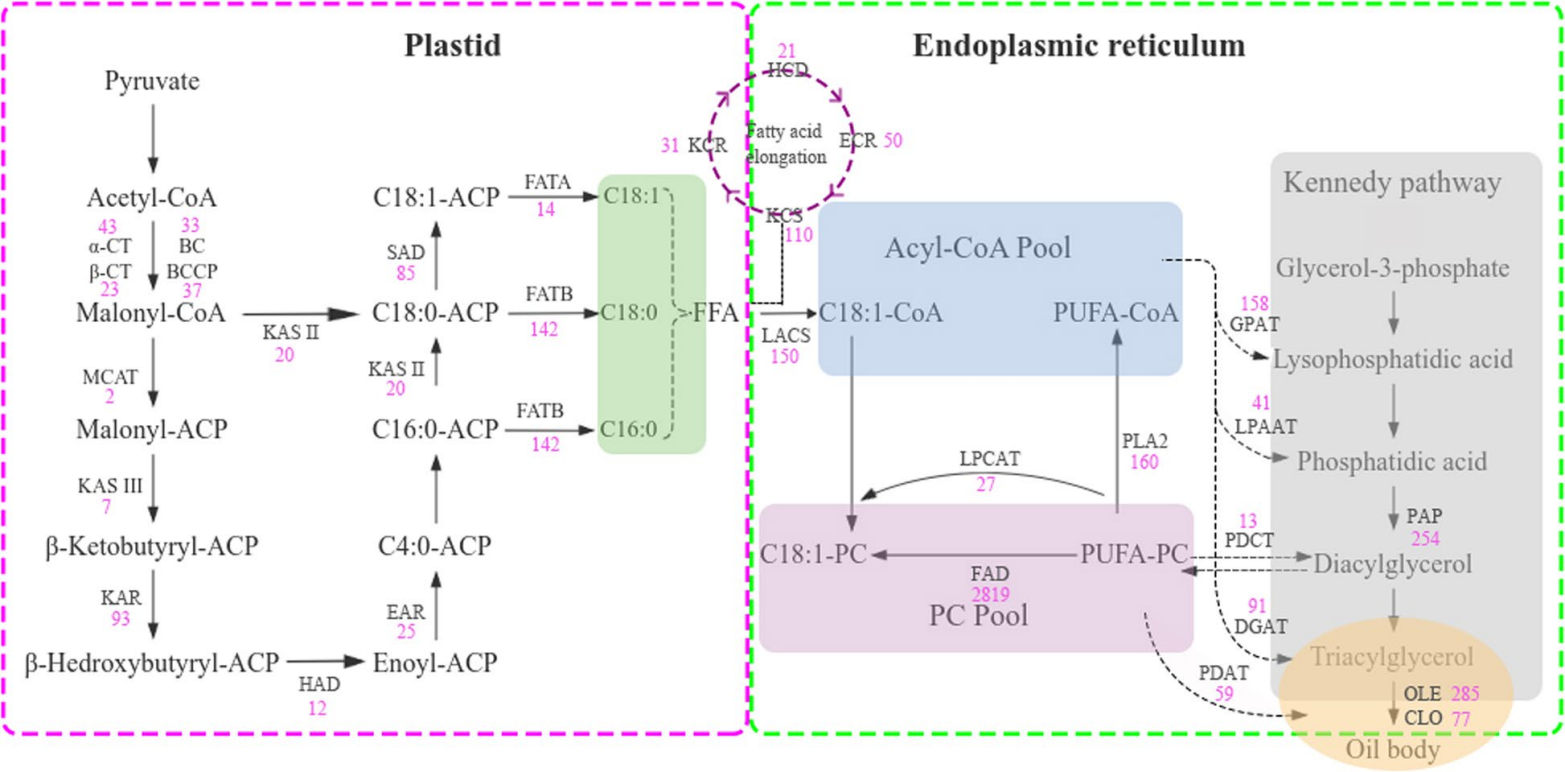
The proposed pathways and genes involved in lipid metabolism in the *Paeonia lactiflora* ‘Hangshao’. This model was developed based on the transcriptome data obtained in this study and information from Meng et al. [5], Zhang et al. [34] and Zhong et al. [35]

Referring to a previously published paper [5], which speculated that *MCAT*, *KASIII*, *FATA*, *SAD*, *FAD*, *DGAT* and *OLE* are the key genes for the biosynthesis of unsaturated fatty acids and oil accumulation in herbaceous peony seeds, we mainly analysed the above seven genes. The malonyl CoA ACP transacylase (MCAT) is the main substrate of the subsequent condensation reaction cycle, converting malonyl-CoA to malonyl-ACP. Only 2 transcripts was identified as *MCAT*. Subsequently, 3-Ketoacyl-ACP synthase III (KASIII) catalyses the conversion of malonyl-CoA to β-ketobutyryl-ACP., and 7 transcripts for *KASIII* was identified. In the initial step, stearoyl-ACP desaturase (SAD) catalyzes the dehydrogenation process, converting C18:0-ACP into C18:1-ACP within the plastid, and 85 transcripts were pinpointed as *SAD*. Then, the fatty acyl-ACP thioesterase A (FATA) converts C18:1-ACP to C18:1, which makes up the free fatty acid (FFA). Only 14 transcripts for *FATA* was identified. Lysophosphatidylcholine acyltransferase (LPCAT) and fatty acid desaturase (FAD) are involved in the biosynthesis of unsaturated fatty acids by facilitating the exchange of unsaturated fatty acids between PC Pool and Acyl-CoA Pool. We identified 27, 2819 transcripts as *LPCAT* and *FAD*, respectively. The synthesis of TAG from glycerol-3-phosphate and acyl-CoA known as the Kennedy pathway. Diacylglycerol acyltransferase (DGAT) catalyses the final step of TAG synthesis, while oleosin (OLE) and caleosin (CLO) are mainly involved in TAG storage. We identified 91, 285 and 77 transcripts as *DGAT*, *OLE* and *CLO*. In most cases, more than one transcript were annotated as the same enzyme, and the transcripts number encoding fatty acid desaturase (FAD) were the most (2,819 transcripts) and followed by oleosin (285 transcripts). The critical steps and key enzymes are shown in Fig. 5. The full names of the individual genes in the figure are detailed in supplementary files (Supplementary table: Table S4).

### Selection and identification of *OLE* and *DGAT* genes utilizing full-length transcriptome

After de-redundancy of the full-length transcriptome database, 10 *OLE* and 5 *DGAT* family genes were identified. OLEs were first found from mustard greens, but the isolate of this protein was originally derived from peanut seeds. Subsequently, a number of plant *OLE* genes were cloned and identified, including mustard, sunflower, cotton, sesame and woody oil plant oil tea [36]. Currently, OLE gene family studies have been conducted in Arabidopsis, peanuts, and some legumes [37–39]. The final step of TAG synthesis to be completed involved the catalysis of *DGAT*. It has been shown that modulation of the expression of *DGAT*, an acyltransferase at the sn-3 locus, can affect the content of ALA. For example, decreasing the expression of *CsDGAT* in *Camelina sativa* can increase the content of ALA in its oil [40]. In order to clarify the relevant protein information of the OLE family of *Paeonia lactiflora*, the physicochemical properties and secondary structural elements of the OLE family members were analyzed by ProtParam and SOPMA. The results showed that the amino acid quantity was between 89–220, the molecular weight was between 9.21kD-23.60kD, and the isoelectric point was between 5.40–10.45. The major secondary structure of other *PlOLEs* is dominated by αlpha-helix, followed by random coil and extended strand apart from *PlOLE1* and *PlOLE10*, while their beta-turn accounting for the least. Compared with OLE in *Arachis hypogaea*, there are also similarity secondary structure, but there are still differences in the ratios [41] (Table 3). Subsequently, we analyzed the basic characteristics of the five identified *PlDGAT* genes, including physicochemical properties and secondary structural elements. Among these PlDGAT proteins, *PlDGAT2* were the smallest *PlDGAT* genes identified, encoding a total of 326 amino acids, while the rest of the genes encoded from 391 to 517 amino acids. The relative molecular weight and isoelectric point analysis of the encoded proteins revealed that their relative molecular weights ranged from 36.68 to 58.79 kDa, and their isoelectric points ranged from 7.18 to 9.28. The aliphatic index is between 78.24 and 103.81, the grand average of hydropathicity (GRAVY) is between -0.431 and -0.261, which means that all five *PlDGATs* are hydrophilic proteins. According to instability index, PlDGAT1, PlDGAT2 and PlWSD2 belong to instability protein, while PlDGAT3 and PlWSD1 belong to stability protein. The secondary structure of them is dominated by αlpha-helix and random coil, followed by extended strand, with minimal to beta-turn (Table 3).

**Table 3:** The physical and chemical properties and secondary structure elements of *OLE* and *DGAT* family members

Gene name	Protein/aa	MW/kD	PI	Aliphatic index	Grand average of hydropathicity (GRAVY)	Instability index	Helix (%)	Extended strand (%)	Random coil (%)	Beta turn (%)
*PlOLE1*	128	13.92	10.45	80.86	-0.082	73.63	17.19	20.31	59.38	3.12
*PlOLE2*	176	19.36	9.76	93.13	0.332	29.58	44.32	21.02	30.11	4.55
*PlOLE3*	136	14.20	9.16	108.31	0.629	19.67	48.53	19.12	27.21	5.15
*PlOLE4*	144	15.07	9.40	101.67	0.237	17.85	40.28	18.75	34.03	6.94
*PlOLE5*	220	23.60	5.40	98.82	0.284	27.63	45.45	16.82	32.27	5.45
*PlOLE6*	166	17.76	9.84	92.23	0.223	28.81	49.40	12.05	30.72	7.83
*PlOLE7*	138	14.45	9.77	108.12	0.416	40.51	50.00	18.12	24.64	7.25
*PlOLE8*	116	11.93	9.60	111.90	0.533	14.82	53.45	14.66	20.69	11.21
*PlOLE9*	123	12.68	10.15	114.96	0.657	33.56	49.59	21.14	19.51	9.76
*PLOLE10*	89	9.21	8.27	124.94	0.654	29.08	28.09	22.47	38.20	11.24
*PlDGAT1*	517	58.79	8.75	103.81	-0.291	43.98	47.20	10.44	39.26	3.09
*PlDGAT2*	326	36.68	9.28	99.54	-0.431	43.17	35.28	20.86	37.12	6.75
*PlDGAT3*	391	41.57	8.61	78.24	-0.261	35.40	33.25	12.02	52.43	2.30
*PlWSD1*	497	56.78	8.97	89.22	-0.299	29.46	38.63	17.10	40.85	3.42
*PlWSD2*	486	55.15	7.18	98.25	-0.271	42.64	39.30	17.49	39.3	3.91

### Conserved Domains and Phylogenetic Analysis of OLEs and DGATs

**Fig. 6:**
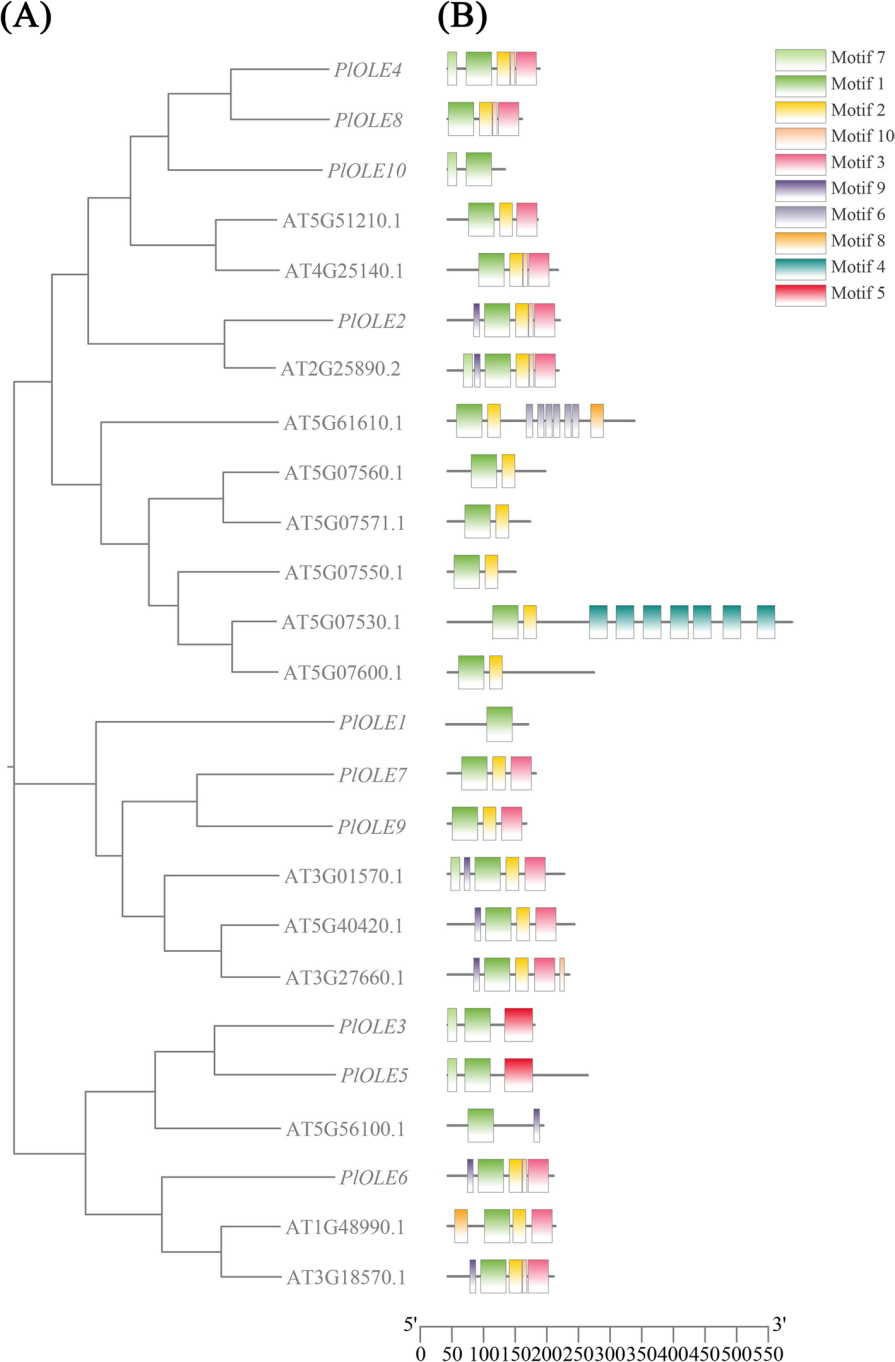
Bioinformatics analysis of *PlOLE* members. **A** Phylogenetic tree of plant OLE homologous proteins. The phylogenetic tree was constructed with neighbor-joining method using MEGA7.0. The statistical reliability of the tree topology was assessed by a bootstrap analysis with 1000 replicates. **B** Schematic diagram of amino acid motifs of OLE protein

**Fig. 7:**
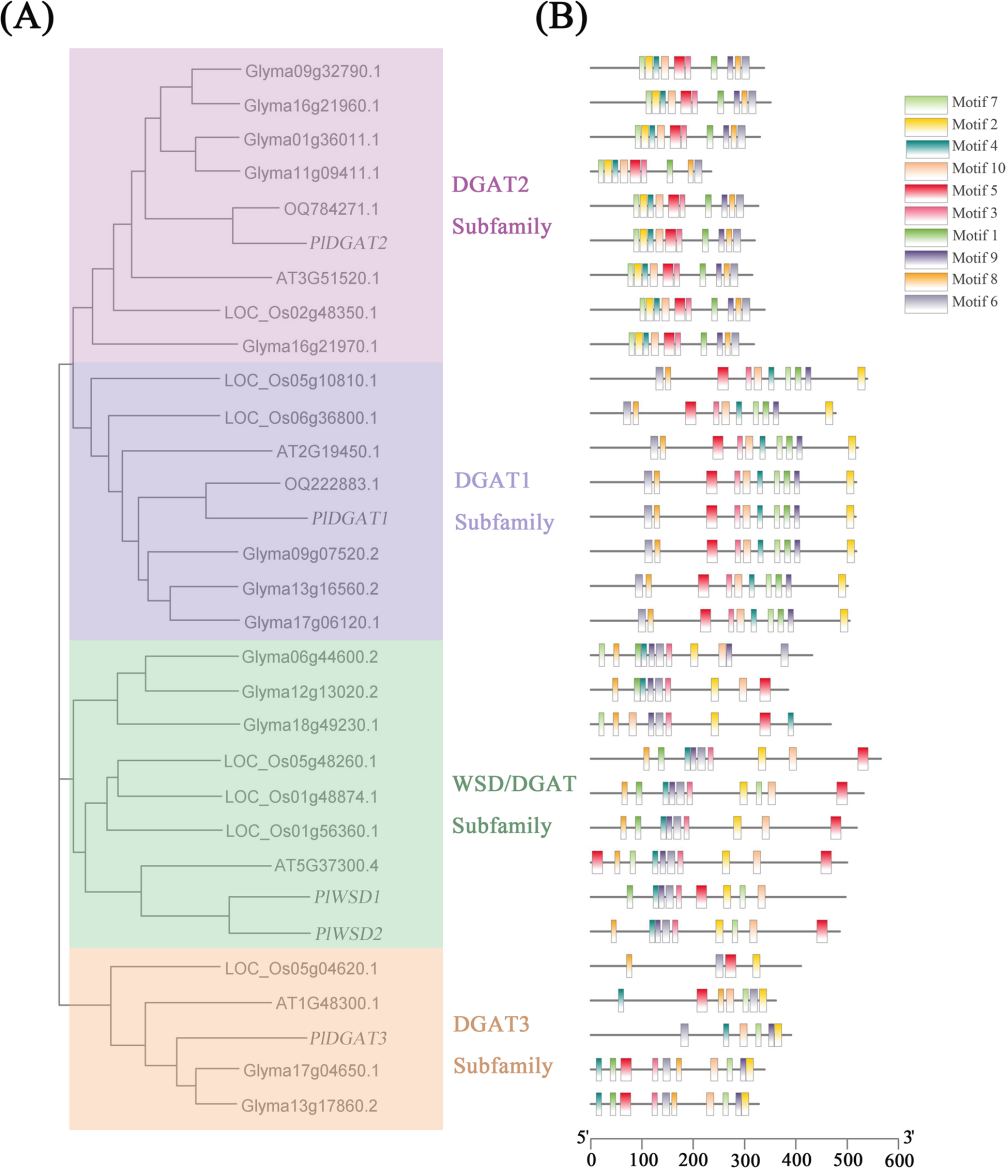
Bioinformatics analysis of *PlDGAT* members. **A** Phylogenetic tree of plant DGAT homologous proteins between *Paeonia lactiflora*, *Arabidopsis*, *Oryza sativa*, *Glycine max* and *Paeonia rockii*. The phylogenetic tree was constructed with neighbor-joining method using MEGA7.0. The statistical reliability of the tree topology was assessed by a bootstrap analysis with 1000 replicates. **B** Schematic diagram of amino acid motifs of DGAT protein

Analysis of protein domains using Pfam and SMART, it was found that these OLE proteins all have conserved structures (Pfam: PF01277), while DGAT was divided into four subfamilies. In addition, we found that the domain distribution of the members of the OLE and DGAT family was roughly the same as that of Arabidopsis, indicating that the conserved domain of the family was positionally conserved across species. However, the functional similarities of these genes are unclear. The genetic evolutionary relationship between *Paeonia lactiflora* and *Arabidopsis thaliana* was analyzed by MEGA7.0 [42] software, and it was found that PlOLE2 were highly similar to Arabidopsis protein (Fig. 6). Each *OLE* gene contains motif 1, at the same time, *PlOLE2*, *PlOLE4* and *PlOLE6* contains the most motif. The genetic evolutionary relationship of DGAT among *Paeonia lactiflora*, *Arabidopsis thaliana*, *Oryza sativa*, *Glycine max* and *Paeonia rockii* was analysed using MEGA 7.0 software (Fig. 7A). To better characterize the PlDGAT family, the motifs in PlDGAT protein sequences were predicted using the MEME online software (Fig. 7B). Based on the number of DGAT domains and the zinc-finger motifs, the putative DGAT proteins could be classified into 4 main groups. It was found that one genes were classified as DGAT1 subfamily, one as DGAT2 subfamily, one as DGAT3 subfamily and two as WSD/DGAT subfamily. Moreover, the conserved domains of each subfamily have a distinct similarity and even contain the same motifs. There were 10 distinct motifs that were identified, and the number of motifs in each DGAT varied between 4 and 10. Most PlDGATs in the same subgroup had similar motif compositions. For example, motif 2–5, 7, and 10 only appeared in DGAT2 subfamily, motif 1–10 only appeared in DGAT2 subfamily, motif 3, 6, 9 occurred in WSD/DGAT subfamily. Interestingly, DGAT1 subfamily was very similar to DGAT2 subfamily, which was consistent with the fact that they have degree of homology (Fig. 7).

### Gene expression analysis

**Fig. 8:**
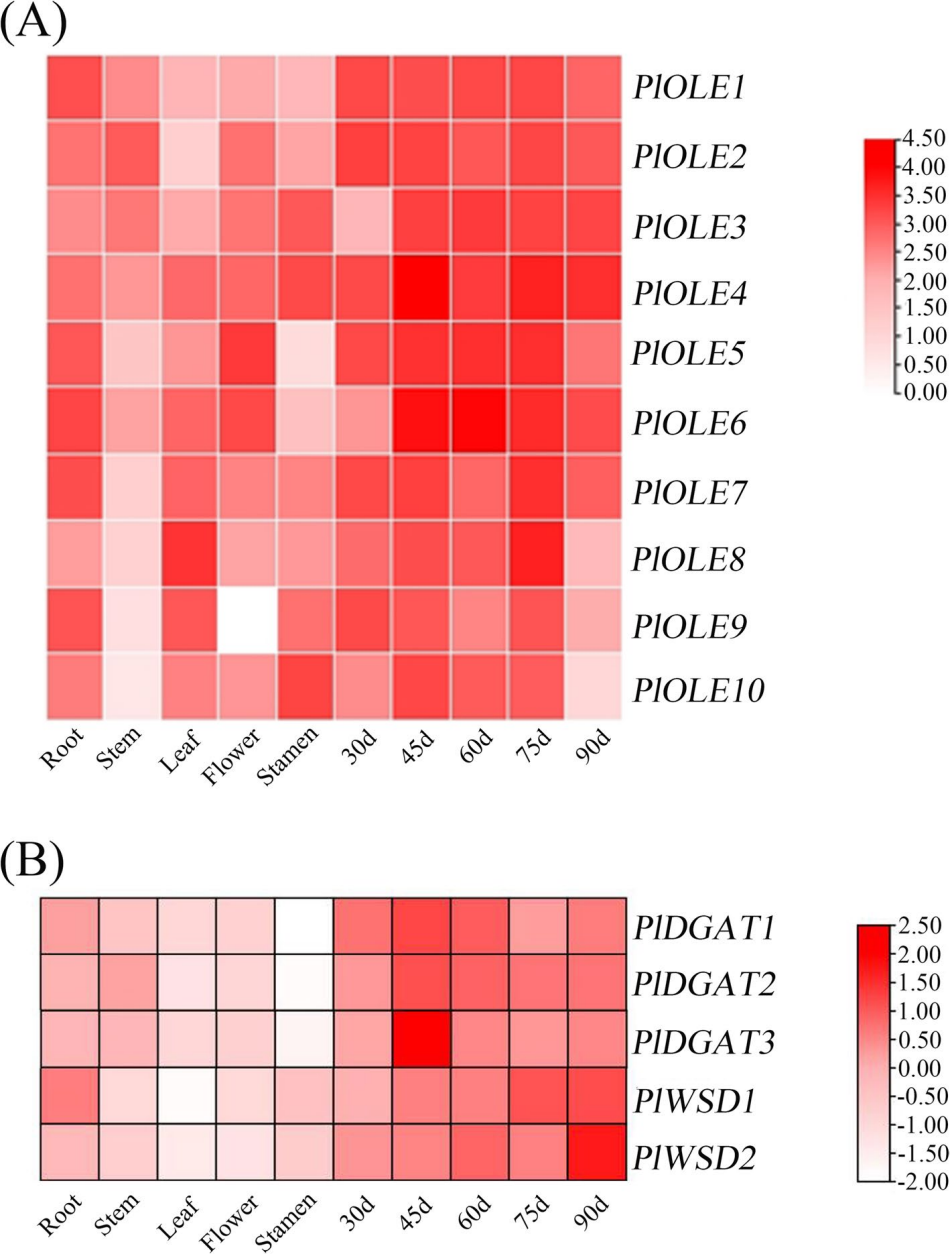
Verification of genes by qRT-PCR. **A** Heatmaps of expression levels of 10 nonredundant *PlOLEs* in the seed of ‘Hangshao’ at different tissues and five developmental stages. **B** Expression heatmap of 5 nonredundant *PlDGATs* in the seed of ‘Hangshao’ at different tissues and five developmental stages. The relative expression value in red indicates the darker the color, the higher the expression level

We analyzed the expression levels of 10 *OLE* and 5 *DGAT* family members on roots, stems, leaves, flowers, stamens, and seeds including 30 days after flower (DAF), 45DAF, 60DAF, 75DAF, and 90DAF to explore whether the expression of *OLE* and *DGAT* genes in different tissues and at different times followed certain expression patterns, and whether these genes were specifically expressed in different tissues. The results obtained are analyzed using TBtools software, and the darker the color, the higher the expression level (Fig. 8). The results showed that the *OLE* gene family was expressed at higher levels in roots, leaves and flowers than in stems and stamens, while the *DGAT* gene family was expressed at higher levels in roots than in stems, leaves flowers, and stamens, and both of them at the highest level in seeds. Most of the genes showed an increasing at first and then tended to decreasing with the time of seed development in *Paeonia lactiflora*. This also indirectly speculates that *OLE* and *DGAT* are involved in the synthesis and accumulation of unsaturated fatty acids by influencing the seed developmental of herbaceous peony,that is beneficial to lay foundations for a more in-depth study of their functions.

## Discussions

*Paeonia lactiflora* as a traditional Chinese flower, because of its large and showy flower is widely loved by people. In recent years, research on herbaceous peony has mainly focused on specific tissues, little research has been done on its full-length transcriptome. With the rapid development of molecular technologies, molecular genetic modification has become a powerful method for flower breeding.

To date, full-length transcriptome information of many species has been obtained through the SMRT technology. For example, for Alfalfa, 21.53 Gb of clean data was obtained using the full-length transcriptome [43], and for maize, 55 Gb of clean data was obtained [44]. It also has been extensively studied in horticulture. For lily, about 36 Gb giant genome was acquired, that will deepen understanding of its bulbil outgrowth [45], for tree peony, a total of 21.27 Gb clean reads were obtained, unveiling potential mechanisms of brassinosteroid-induced delayed flowering in peony [46], for *Camellia oleifera*, *cv. Min 43* (M43) contained 41.49 Gb clean reads, and *cv. Hongguo* (HG) contained 38.99 Gb clean reads, help to unveil potential mechanisms of triacylglycerol degradation during seed desiccation [47]. In this study, a total of 10,187,282 subreads were obtained from 41.35 Gb of data using SMRT sequencing technology. We clustered the corrected transcript sequences according to the 95% similarity among the sequences, then remove redundancy and finally obtained 1,335,148 specific transcripts. 484,931 ROI sequences were obtained and 1,455,771 FLNC transcripts for further functional annotation, CDS and transcription factor prediction, SSR analysis, and lncRNA identification. The COG database annotated genes related to lipid transport and metabolism, while metabolic processes are the terms that account for a relatively high proportion of the GO annotation system. We then performed structural analysis and functional annotation of these transcripts, which provided an important database for further molecular studies on herbaceous peony. Since *Paeonia lactiflora* does not yet have a wide-genome, it is particularly important to study the molecular mechanisms of peony through a full-length transcriptome. A large number of full-length transcripts were obtained through the full-length transcriptome, which provided more information for the molecular mechanism of subsequent herbaceous peony growth and development, and also laid an important foundation for molecular breeding.

In recent years, many studies have found that the seed fruiting rate, oil content and unsaturated fatty acid content of 'Hangshao' have a well performance, and close to the *Paeonia suffruticosa* variety 'Fengdan', which is expected to be developed into a new type of oil plant [5]. In order to avoid a huge waste, we have carried out extensive studies on its fatty acid biosynthesis pathway, since the seeds of *Paeonia lactiflora* are rich in unsaturated fatty acids. Fats and oils are the main source of energy metabolism in living organisms, mainly synthesised in the form of TAG in plants [48]. It was found that oleosin regulate lipid metabolism during seed germination [49], diacylglycerol acyltransferase (DGAT) is considered to be the key enzyme for the last step of triacylglycerol synthesis and the only rate-limiting enzyme, both of which play key roles in the biosynthesis and storage of TAG. Consequently, 4,905 genes in pathways related to lipid metabolism were annotated with transcriptome sequencing. A recent study in *Paeonia lactifolra* found that the comparative transcriptome analysis of herbaceous peony at different development stages provides an effective way to study gene differential expression patterns and dissect oil synthesis candidate genes [5]. In our study, we had identified and analysed 10 *PlOLEs* and 5 *PlDGATs* using the full-length transcriptome data of *Paeonia lactiflora* after de-redundancy, which is of significance in studying lipid metabolism in this species.

Oleosin protein is a structural protein that is first isolated and identified on seed oil bodies [50]. It consists of three parts, the N-terminal hydrophilic domain, the hydrophobic central structural domain and the most conservative hydrophobic hairpin zone (about 72 residues) and the C-terminal α-helical structural domain [51]. Amphiphilic oleosins are able to stabilize intracellular hydrophobic triglycerides (TAG) by inserting their hairpin regions into the oil body and exposing their N- and C-terminal hydrophilic regions [52]. To data, oleosin protein have been successively reported in different oil crops, such as soybean [53], vernicia tree [54] and peanut [55] etc. In *Cyperus esculentus*, 9 *OLE* and 21 *CLO* genes were identified, which can be provided a reference for the development of strategies to improve oil content of *C. esculentus* tubers [56]. In *Carthamus tinctorius*, 8 putative *OLE* genes were identified from the genome database, providing a way of elucidating the intricate mechanisms of oil body synthesis [57]. Using the full-length transcriptome we identified 10 *PlOLEs*, and the number of genes was not significantly different from the other species, proving the reliability of the results. Protein physicochemical properties and phylogenetic analysis showed that they also share certain similarities. The results indicated that the amino acid quantity was between 89–220, the molecular weight was between 9.21kD-23.60kD, and the isoelectric point was between 5.09–10.45. Phylogenetic and motif analysis showed that ten oleosin proteins are homologous to Arabidopsis and each of them contains motif 1, indicating that they are highly conserved here. We found that the *OLE* involved in the TAG assembly were highly expressed at the 45d of seed development, concomitant to the active oil biosynthesis in this period. Overall, we found that the expression patterns of 10 *OLEs* verified by qRT-PCR at higher levels in seed than in other tissues, and showed a trend of increasing first and then decreasing with the development of seeds (Fig. 8A).

DGAT is responsible for transferring acyl of acyl CoA to DAG and plays a key role in controlling lipid synthesis [58]. Many studies have been conducted to increase TAG production and fatty acid content by manipulating the *DGAT* gene. Four subfamilies of DAGT enzymes have been identified in plants, DGAT1, DGAT2, DGAT3 and WSD/DGAT, respectively. For example, in Arabidopsis and most oilseed crops, *DGAT2* are generally specialized in catalyzing the acylation of unusual fatty acids onto DAG molecule, and hence responsible for the content of TAG containing unusual fatty acids, whereas *DGAT1* was regarded as the key player in determining oil content in seeds. However, in peanut, all three *DGATs* (*DGAT1*, *DGAT2*, and *DGAT3*) are involved in TAG synthesis [59]. In *Paeonia rockii*, *PrDGAT3* is essential in TAG synthesis and has a substrate preference for polyunsaturated fatty acids, especially LA and ALA. A recent study in *Zea mays*, overexpression of *DGAT1* not only increased the oil content of maize seeds, but also altered the composition of seed lipids [60]. In this study, the transcript of *DGAT3* were more abundant than *DGAT2* in herbaceous peony (Fig. 8B), in congruence with previous studies [61]. In *Physaria fendleri*, four *PfDGATs* were identified [61]. Through genome identification analysis, 7, 7, 9, and 10 members of the DGAT family were identified in maize, rice, sorghum, and foxtail millet, respectively [62]. We identified 5 *PlDGATs* based on full-length transcriptome. The physical and chemical properties indicated that the protein numbers was range from 326 to 517, the molecular weight was range from 36.68kD to 58.79kD, and the isoelectric point was range from 7.18 to 9.28. According to secondary structure analysis, three of them belong to unstable proteins, and four proteins mainly dominated by irregular. Phylogenetic and motif analysis showed that 5 *PlDGATs* were homologous to soybean, rice and *Arabidopsis*, of which *PlDGATs* were distributed in four subfamilies, the composition of motifs of the same subfamily is essentially the same. The gene structures of DGAT members of different subfamilies differed significantly, whereas the distribution of motif structures among members of the same subfamily was basically the same, suggesting that different DGAT subfamilies have a high degree of conservatism while undergoing parallel evolution, and that the generation of such differences in gene structure may be a conserved mode of evolution for the DGAT gene family. *PlDGAT1*, *PlDGAT2* and *PlDGAT3* were highly expressed at 45d of seed development, in congruence with the accumulation rate of fatty acids in herbaceous peony seeds, while the expression levels of *PlWSD1* and *PlWSD2* generally increase and reach the highest level in the late stage of seed development. The expression pattern of *PlDGATs* at higher level in seeds than in other tissues. The result is consistent with *PlOLEs*, indicating that they play an important role in seed development period.

In conclusion, *PlOLEs* and *PlDGATs* had a significant response in the initial period of seeds development and a higher expression level in seeds compared with other tissues. In general, this finding significantly improves our knowledge of the biosynthesis pathways of lipid metabolism, this study provides a basis for further research on the molecular functions and regulatory mechanisms of *PlOLEs* and *PlDGATs*.

## Conclusions

In this study, we used the full-length transcriptome to reveal the molecular mechanisms of herbaceous peony, providing a basis for subsequent research on the herbaceous peony. We identified and analysed genes associated with the biosynthesis pathway of lipid metabolism, it was found that lipid metabolism is completed in plastid and endoplasmic reticulum, *OLE* and *DGAT* are involved in the Kennedy pathway. In addition, we identified 10 *PlOLE* and 5 *PlDGAT* family members and analyzed their physicochemical properties, conservative protein motifs, and phylogenetic trees. Finally, we analyzed the expression patterns of *PlOLEs* and *PlDGATs* to help us to better understand the functionality which may play roles in lipid metabolism pathways.

## Materials and methods

### Plant materials

**Fig. 9:**
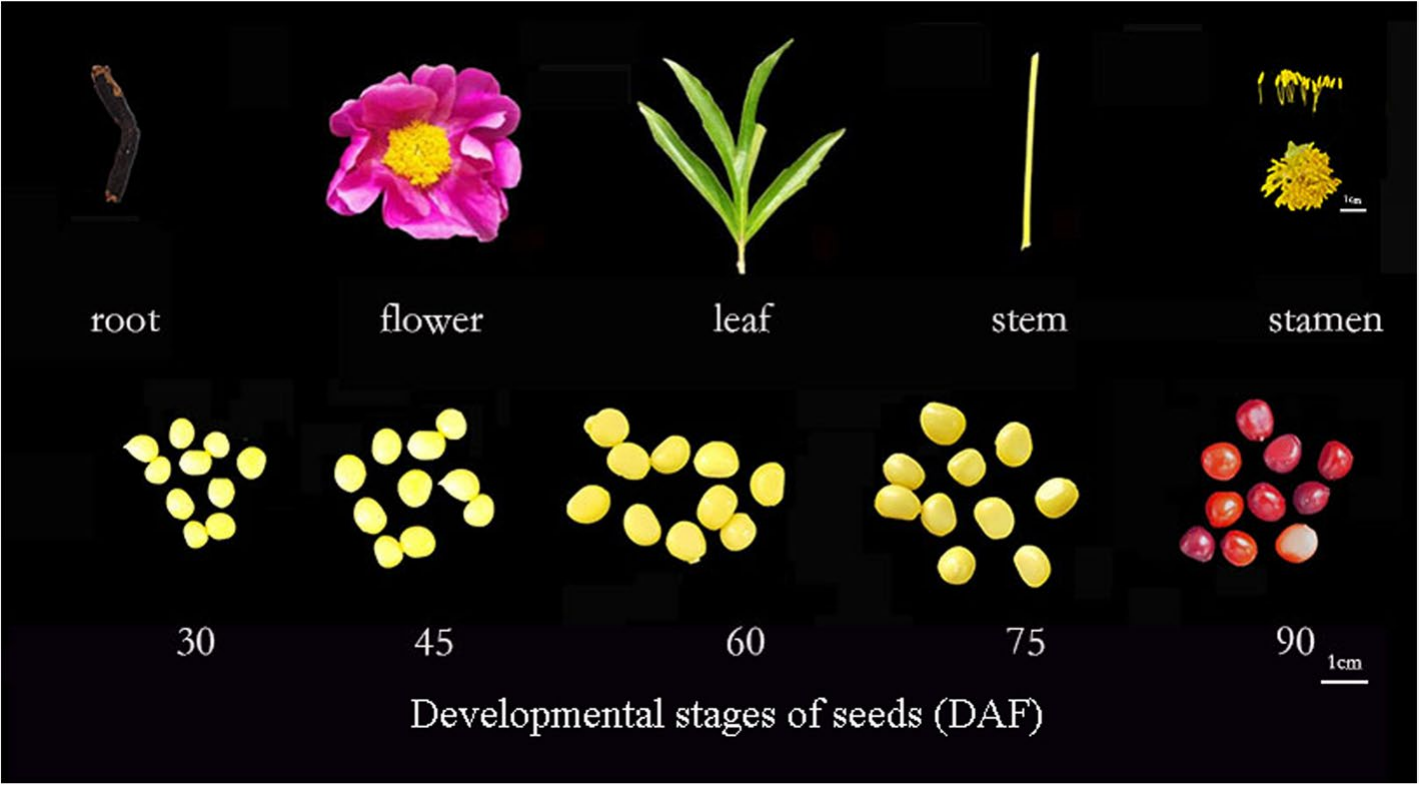
The tissues of *Paeonia lactiflora* Pall. used in this study

The plant materials used in this experiment was ‘Hangshao’ variety of *Paeonia lactiflora* from the germplasm repository of college of Horticulture and Landscape Architecture, Yangzhou University, Jiangsu Province (32°23′31’N, 119°24′50’E).According to our previous experiment, young leaves, stems, roots, flowers, seeds which are collected 30, 45, 60, 75, and 90 days after flowering, and stamen of ‘Hangshao’ [5] (Fig. 9). Seeds, leaves, flowers, stamen, roots and stems used for qRT-PCR from the same herbaceous peony plant.

### RNA sample preparation

There were three biological replicates for each sample, and stored in liquid nitrogen for RNA extraction. RNA was extracted from plant tissue using CTAB methods. To ensure the accuracy of the sequencing data, all RNA samples quality were measured using a NanoDrop 2000 spectrophotometer (Thermo Fisher Scientifc, Waltham, MA, USA). The RNA integrity was checked using an Agilent 2100 bioanalyzer (Agilent Technologies, Santa Clara, CA, USA), which included RIN, 28S, 18S and 5S peaks. Electrophoresis was used to detect whether the RNA samples contained gDNA contamination and assess RNA quality by identifying the ribosomal bands.

### Library construction and SMRT sequencing

After RNA quality testing, we first mixed an equal amount of high-quality RNA from different tissues of ‘Hangshao’ and then mixed it into a sample bank [63]. Extract all digested RNA samples, thermal degeneration opens its secondary structure, enrich mRNA using oligo (dT) magnetic beads. The divalent cations were applied to manage the fragmentation under elevated temperatures. The first strand of cDNA was synthesized using the UMI base PCR cDNA Synthesis Kit (BGI), and the first strand of cDNA was amplified by PCR to synthesize the double-strand cDNA. Prepare the reaction system, the temperature reaction for a certain time, repair the double-strand cDNA end, and add an A base at the 3' end, prepare the linker to connect the reaction system, the temperature reaction for a certain time, so that the linker and cDNA are connected, the ligation product is amplified. After the PCR product is denatured into a single strand, the cyclization reaction system is prepared, the temperature response is a certain time, the single-stranded ring product is obtained, and the final library is obtained after digesting the linear DNA molecules that have not been cyclized.. The libraries were evaluated quantitatively by a Qubit2.0 DNA kit (Life Technologies, China), size of the libraries was detected by Agilent 2100.

### PacBio Iso-Seq data processing and bioinformatics analysis

After sequenced by PacBio sequel, large number of Circular Consensus Sequencing (CCS) reads were obtained. Reads of insert (ROI) was identified and classified into full-length non-chimeric (FLNC) and non-full-length (nFLNC) reads. The full-length and non-full-length fasta files produced were then fed into the cluster step, which performs isoform-level clustering Interative Clustering and Error Correction (ICE), similar sequences were clustered into clusters, each of which yields a consensus isoform, followed by final Arrow polishing. The final Isoform sequence is obtained using CD-HIT [64] software for de-redundancy. The resulting transcript sequence can be directly used for subsequent analysis, gene families, CDS, TF, SSR, lncRNA and other analyses.The TransDecoder (https://transdecoder.github.io) software is used to identify the longest Open Reading Frames (ORFs), and then searching for Pfam protein homologous sequences by blast comparing SwissProt (http://ftp.ebi.ac.uk/pub/databases/swissprot) and Hmmscan (http://hmmer.org) to predict the coding regions. All transcription factors (TFs) were identified by using the Plant Transcription Factor Database (Plant TFDB,http://planttfdb.gao-lab.org/index.php?sp=Zma) [65] and GRASSIUS (https://grassius.org/tfomecollection.php) [66]. If a gene appears in any of databases, the gene is considered as TF and the corresponding transcript of the TF encoding gene is retrieved. Additionally, full-length transcriptome has been helpful for marker discovery for simple sequence repeats. We used MISA (http://pgrc.ipkgatersleben.de/misa/misa.html) to identify SSRs. We also screened transcripts with coding potential to obtain predicted lncRNA. In this study, the most widely used coding potential analysis methods to predict lncRNA in transcripts, including four methods: CPC analysis [31], CNCI analysis, Pfam protein structure and analysis, and txCdsPredict analysis.

### Functional annotation and enrichment analysis

We used BLAST to combine the obtained sequence of non-redundant transcripts with NR (NCBI non-redundant protein sequences database), Nt (http://www.ncbi.nlm.nih.gov), SwissProt (http://www.ebi.ac). uk/swissprot), GO (http://www.geneontology.org), KOG (https://mycocosm.jgi.doe.gov/ help/kogbrowser.jsf), Pfam (http://pfam.xfam.org/) and KEGG (http://www.genome.jp/kegg) databases, to get annotation information for the transcript. The results of enrichment analysis were visualized by the enrichplot and ggplot2 packages.

### Analysis of the *OLE* and *DGAT* genes family in *Paeonia lactiflora*

To classify the *PlOLE* and P*lDGAT* genes in *Paeonia lactiflora*, Cluster X 2.0.12 software (http://www.cluster-x.org/) was applied for multiple sequence alignment by using protein sequences of Arabidopsis. SMART (http://smart.embl-heidelberg.de/) and CDD (https://www.ncbi.nlm.nih.gov/Structure/cdd/wrpsb.cgi) were used to manually confirm whether the candidate genes were *PlOLE* and *PlDGAT* genes. Their serial information is detailed in the Supplementary Table S5. To construct the phylogenetic tree, neighbor-joining (NJ) method was used by MEGA7.0 software, and bootstrap values were set as 1000 bootstrap replicates [42]. The conserved motifs of the *PlOLE* and *PlDGAT* sequences were identified by the MEME program (https://meme-suite.org/meme/), and the parameters were set as a maximum of 10 motifs and an optimum motif width of 6–200 amino acid residues [67]. The conserved domains were visualized using the TBtools software.

### Validation of gene expression by Quantitative Real-Time PCR (qRT-PCR)

Each plant tissue is represented by three biological replicates and three technical replicates. Extract RNA from plant roots, stems, leaves, flowers, stamens, and seeds which are collected 30, 45, 60, 75and 90 days after flowering by using the TaKaRa Mini Best Plant RNA Extraction Kit (TaKaRa, Japan). Then use PrimeScript ®RT reagent Kit (TaKaRa, Japan) with gDNA Eraser (Perfect Real Time) the kit reverses the total RNA of the sample into cDNA [68]. NovoStart® SYBR qPCR SuperMix Plus kit (Novoprotein, China) was used for qRT-PCR analysis on the Bio-Rad CFX Manager V1.6.541.1028 software. The *PlActin* (JN105299) gene was used as an internal reference for this experiment and the expression level of this reference gene was stable in all organs of *Paeonia lactiflora*. The primers were designed using Primer Premier 5, and all primers were listed in table (Supplementary table: Table S6). The relative expression levels of the target genes were calculated using the 2^−∆∆Ct^ method, and the data were analyzed by the TBtools software.

### Supplementary Information

Supplementary Material 1.

## Data Availability

The datasets generated or analysed during the current study are available in the main paper and supplementary information files, The raw reads are available in the Sequence Read Archive (SRA) database of the National Center for Biotechnology Information (NCBI) under accession number PRJNA1064234.

## Abbreviations

FAD: Fatty acid desaturase
OLE: Oleosin
PAP: Phosphatide phosphatase
FAD-delta-12: Delta(12) fatty acid desaturase
PLA2: Phospholipase A2
GPAT: Glycerol-3-phosphate acyltransferase
LACS: Long-chain acyl-CoA synthetase
FATB: Fatty acyl-ACP thioesterase B,
KCS: 3-Ketoacyl-CoA synthase
KAR: 3-Ketoacyl-ACP reductase
DGAT: Diacylglycerol acyltransferase
SAD: Stearoyl-ACP desaturase
CLO: Caleosin
PDAT: Phospholipid:diacylglycerol acyltransferase
KAS: 3-Ketoacyl-ACP synthase
ECR: Enoyl-CoA reductase
α-CT: Carboxyltransferase subunit alpha
LPAAT: Lysophosphatidic acid acyltransferase
BCCP: Biotin carboxyl carrier protein
BC: Biotin carboxylase
KCR: Ketoacyl-CoA reductase
LPCAT: Lysophosphatidylcholine acyltransferase
EAR: Enoyl-ACP reductases
β-CT: Carboxyltransferase subunit beta
HCD: Hydroxyacyl-CoA dehydratase
FATA: Fatty acyl-ACP thioesterase A
PDCT: Phosphatidylcholine:diacylglycerol cholinephosphotransferase
HAD: Hydroxyacyl-ACP dehydratase
MCAT: Malonyl-CoA:ACP transacylase
